# A Three-Reagent “Green” Paper-Based Analytical Device for Solid-Phase Spectrometric and Colorimetric Determination of Dihydroquercetin

**DOI:** 10.3390/s22082893

**Published:** 2022-04-09

**Authors:** Vladimir V. Apyari, Aleksei A. Furletov, Vyacheslav I. Kalinin, Stanislava G. Dmitrienko, Yury A. Zolotov

**Affiliations:** 1 Department of Chemistry, Lomonosov Moscow State University, Leninskie Gory, 1/3, 119991 Moscow, Russia; aleksei_furletov@mail.ru (A.A.F.); workm2k@gmail.com (V.I.K.); dmitrienko@analyt.chem.msu.ru (S.G.D.); zolotov@analyt.chem.msu.ru (Y.A.Z.); 2 Kurnakov Institute of General and Inorganic Chemistry, Russian Academy of Sciences, Leninsky Avenue, 31, 119991 Moscow, Russia

**Keywords:** microfluidic paper-based analytical devices, antioxidants, flavonoids, dihydroquercetin, optical sensors, solid-phase spectrophotometry, colorimetry

## Abstract

Microfluidic paper-based analytical devices (µPADs) represent one of the promising green analytical strategies for low-cost and simple determination of various analytes. The actual task is the development of such devices for quantitation of antioxidants, e.g., flavonoids. In this paper, possibilities of a novel three-reagent µPAD including silver nitrate, 4-nitrophenyldiazonium tetrafluoroborate, and iron(III) chloride as reagents are assessed with respect to the determination of dihydroquercetin. It is shown that all the three reagents produce different colorimetric responses that can be detected by a mini-spectrophotometer–monitor calibrator or by a smartphone. The method is applicable to direct measuring high contents of dihydroquercetin (the linearity range is 0.026–1 mg mL^−1^, and the limit of detection is 7.7 µg mL^−1^), which is favorable for many dietary supplements. The analysis of a food supplement was possible with the relative standard deviations of 9–26%, which is satisfactory for quantitative and semiquantitative determinations. It was found that plotting a calibration graph in 3D space of the three reagents’ responses allows us to distinguish dihydroquercetin from its close structural analogue, quercetin.

## 1. Introduction

Microfluidic paper-based analytical devices (µPADs) have attracted a lot of attention due to the wide prospects for their application in many areas of science and technology—in medicine, diagnostics, environmental monitoring, food processing, agriculture, and other fields [1,2,3,4,5,6,7,8,9,10,11]. The most important advantages of µPADs are the absence of any pumps, small sample volume, rapid analysis, an ability of naked eye detection, low cost, ease of use, compactness, and biodegradability. The hydrophilic fibrillar structure of paper not only allows for the solution to move without the use of a pump, but also provides a filtering ability, which, in some cases, makes it possible to use such systems for samples with a complex matrix [12]. Often, µPADs are needed as a screening analytical tool to identify samples that should be subjected to a deeper study.

One of the actual practical applications of µPADs is the determination of biologically active substances. Antioxidants, such as flavonoids, are among them. To date, a number of analytical methods have been developed for the determination of flavonoids, such as HPLC [13,14,15,16,17,18], gas chromatography [19,20], capillary electrophoresis [21,22,23], voltammetry [24,25,26], and spectrophotometry [27,28,29,30,31].

The widespread occurrence and pronounced biological activity of flavonoids make it important to develop simple and inexpensive methods for their determination, suitable for large-scale use. At the same time, the variety of emerging practical problems stimulates the search for options of simple optimization of the analytical system architecture for a specific analysis, which, in our opinion, can be implemented by combining its separate, pre-produced parts. This idea has found its implementation in a concept of composable paper-based analytical devices, which can be assembled in a required architecture using several pre-made paper parts [32].

Within this concept, we present here a novel three-reagent paper-based analytical device for solid-phase spectrometric and colorimetric determination of dihydroquercetin.

## 2. Materials and Methods

### 2.1. Reagents

The following reagents were used: silver nitrate (PZTsM-Vtormet, Moscow, Russia, analytical grade), sodium hydroxide (Reachim, Moscow, Russia, reagent grade), iron(III) chloride (Acros Organics, 98%), 4-nitrophenyldiazonium tetrafluoroborate (4-NPD) (synthesized according to [33]), dihydroquercetin (Flamena, Moscow, Russia, analytical grade), and quercetin dihydrate (Sigma-Aldrich, St. Louis, MO, USA, ≥98% (HPLC)). Working solutions of flavonoids were prepared using ethanol (Komponent-Reactive, Moscow, Russia, reagent grade). To check the interfering effect, salts were used: NaCl (reagent grade), KCl (reagent grade), Na_2_SO_4_ (reagent grade), and Ca(NO_3_)_2_ (reagent grade).

Microfluidic systems were assembled from pre-made paper elements, which were Whatman Grade 1 filters with a diameter of 10 mm, on double-sided tape (Unibob).

### 2.2. Instruments

Diffuse reflectance spectra in the visible spectral range were recorded on an i1Pro2 mini-spectrophotometer (X-Rite, Grand Rapids, MI, USA). Photographs of the samples were taken using an iPhone 10 smartphone (Apple, Cupertino, CA, USA). Chromatograms of the samples were recorded using a Tsvet Yauza liquid chromatograph (NPO Khimavtomatika, Voronezh, Russia) with an amperometric detector (E = 1.3 V). A chromatographic column Luna C18 (Phenomenex, Torrance, CA, USA) was used. The mobile phase consisted of acetonitrile: 0.1% aqueous solution of H_3_PO_4_ (25:75). The sample volume was 20 μL; the sample was injected using a dispenser loop. The flow rate was 0.4 mL min^−1^. Deionized water was obtained using a Simplicity purification system (Millipore, Burlington, MA, USA). A mechanical shaker was used for mixing. The drying of samples was carried out on a household electric hot plate.

### 2.3. Procedures

To construct a microfluidic system, double-sided tape was attached to a transparent polymer plate. Then, three paper elements were appropriately placed to prepare the detection zones, and the reagents were applied to them as follows: (1) 40 μL of 20 mM FeCl_3_ solution in ethanol; (2) 40 μL of 10 mM 4-NPD solution in 80% ethanol; and (3) 40 μL of 10 mM AgNO_3_ aqueous solution followed by drying on an electric hot plate at ~80 °C and addition of 40 μL of 1 mM NaOH aqueous solution. The microfluidic system was dried at ~80 °C. Then, a loading paper zone was fixed on the scotch tape between all the three detection zones so that the intersection of the loading zone with each detection zone was about 1 mm.

The as-prepared microfluidic system was used for the determination of dihydroquercetin as follows. An 80 µL aliquot of dihydroquercetin solution in ethanol (from 0 to 1.2 mg mL^−1^) or an analyzed sample was applied to the loading zone. Then, the system was dried in air.

Finally, diffuse reflectance spectra of each colored detection zone were recorded using the mini-spectrophotometer as follows. The microfluidic system was placed on a dense paper sheet, the target of the mini-spectrophotometer, previously calibrated against a standard white sample, was placed on the detection zone, and the reflection coefficients were measured.

The spectra were plotted in coordinates of the Kubelka–Munk function (F) versus wavelength (λ, nm). F values were calculated using the formula F(R) = (1 − R)^2^/(2R), where R is the diffuse reflection coefficient at a given wavelength. In some cases, normalized diffuse reflectance spectra were calculated dividing the Kubelka–Munk function meanings (F) at each λ by the maximum of them (F_max_); they were designated as F/F_max_. To obtain a function proportional to the concentration of dihydroquercetin, ΔF = F − F_0_ was calculated; here, F_0_ is the Kubelka–Munk function measured for the blank.

## 3. Results and Discussion

**Figure 1:**
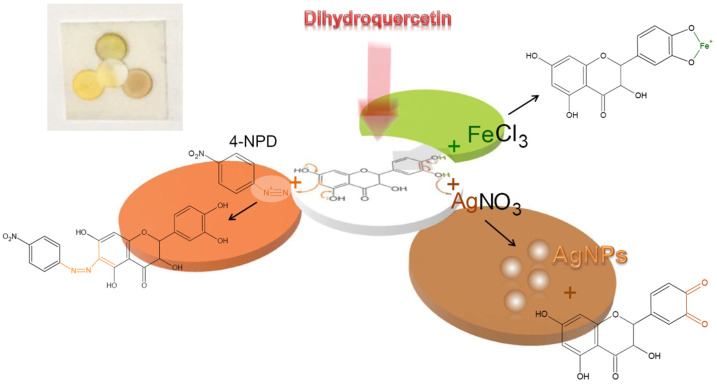
Schematic representation of the experimental µPAD design for the determination of dihydroquercetin and supposed colorimetric reactions within it. The insert is a photo of the µPAD after application of dihydroquercetin.

The concept of composable µPADs [32] was applied in this study to develop a method for the determination of dihydroquercetin. This concept implies designing a microfluidic system of a certain architecture for solving a particular analytical problem from ready-made elements that are fixed on an adhesive substrate. This approach allows for easy and quick assembling and changing the system architecture, depending on the task. Cut paper parts do not require waxing or 3D printing. Unused items can be saved for later applications, reducing the cost of analysis. For the manufacturing of paper elements, pre-fabricated Whatman Grade 1 disc filters with a diameter of 10 mm were used. Double-sided tape on a polymer plate was used as an adhesive substrate. Paper elements were fixed on it to provide three independent microfluidic branches with a common central loading zone. A slight overlap (about 1 mm) was made between the loading zone and each of the three detection zones to ensure the transition of liquid from one element to another (Figure 1).

### 3.1. Choice of the Reagents for Determination of Dihydroquercetin

When choosing reagents for the colorimetric detection of dihydroquercetin using the proposed µPADs, its chemical and structural features were taken into account. They are the presence of acidic phenolic hydroxyls capable to form chelate complexes, an aromatic system with π-donor radicals capable of electrophilic substitution reactions, and the pronounced reducing properties of this compound (Figure 1).

First, due to the polyphenolic nature of dihydroquercetin, iron(III) chloride was chosen as one of the reagents for its colorimetric determination. Iron(III) in the reaction with phenols forms complex compounds colored in violet, blue, or green [33]. The maximums of their absorption band, as a rule, lie in the range of 500–600 nm.

Secondly, phenols easily participate in azo-coupling reactions with aromatic diazonium salts, forming intensely colored products [33,34]. Often, 4-nitrophenyldiazonium, as its tetrafluoroborate ionic associate (4-NPD), is used as a diazo-component during photometric determination of phenols [35,36]. The advantage of 4-NPD is its stability in solid form and relative stability in solutions. The presence of phenolic fragments in the dihydroquercetin molecule also determines its participation in the azo-coupling reaction with 4-NPD, resulting in the extension of the delocalized π-electron system and a bathochromic shift of a spectral band.

Finally, another characteristic feature of dihydroquercetin is its pronounced antioxidant properties. Therefore, silver nitrate, which participates in a redox reaction with dihydroquercetin, was chosen as a third reagent in this µPAD. As reported earlier [37,38], flavonoids reduce silver nitrate to silver nanoparticles with an intense surface plasmon resonance (SPR) band. This property of silver nanoparticles was used to detect dihydroquercetin in this study. In contrast to the absorption bands of the flavonoids themselves, located in the UV spectral range (280–380 nm), the maximum of silver nanoparticles SPR band lies in the visible spectral range (~420 nm), which can be successfully used for the detection using paper microfluidic systems even with a naked eye. Since the reaction with silver nitrate takes place in a basic medium, alkali must be used as an additional reagent in this case.

### 3.2. Determination of Dihydroquercetin Using the Three-Reagent µPAD and Diffuse Reflectance Spectroscopy

#### 3.2.1. Analytical Responses

**Figure 2:**
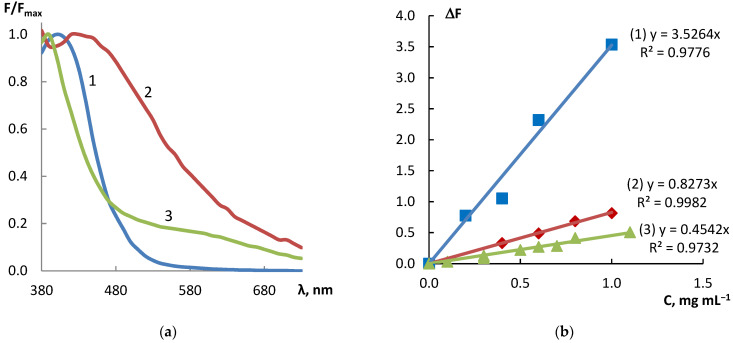
(**a**) Normalized diffuse reflectance spectra of products of interaction between 4-NPD (1), AgNO_3_ + NaOH (2), FeCl_3_ (3), and dihydroquercetin recorded on the µPAD detection zones; (**b**) corresponding calibration graphs plotted at 400, 420, and 500 nm, respectively, with their equations and squared correlation coefficients.

**Figure 3:**
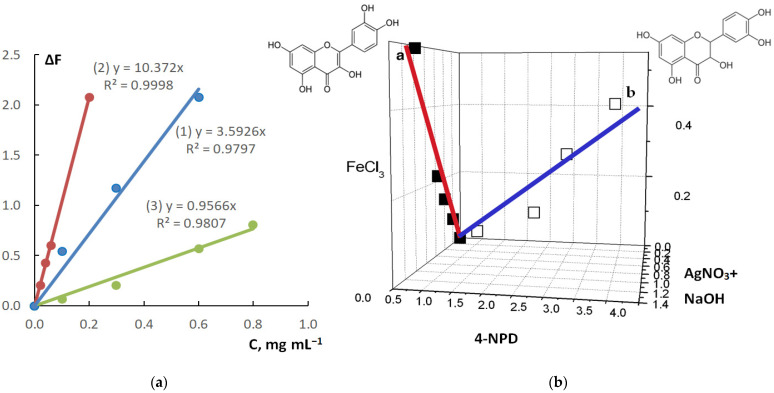
(**a**) Calibration graphs based on the interaction between 4-NPD (1), AgNO_3_ + NaOH (2), FeCl_3_ (3), and quercetin recorded on the µPAD detection zones (with their equations and squared correlation coefficients); (**b**) 3D chart of the corresponding detection zones responses regarding quercetin (a■) and dihydroquercetin (b□).

The normalized diffuse reflectance spectra of products of interaction between the above-mentioned three reagents and dihydroquercetin recorded on the µPAD detection zones are represented in Figure 2. It can be seen that the products have different spectral characteristics including both the position of the absorption band maximum and its shape. Together with different principles of interaction between these reagents and dihydroquercetin, which was discussed above, it stipulates good prospects of the proposed µPAD in multi-responsive methods of substances identification and determination. To check this supposition, we compared responses of µPAD regarding quercetin, which differs from dihydroquercetin by one more double bond (Figure 3). The corresponding calibration graphs for quercetin are depicted in Figure 3a. A comparison of these graphs with those plotted for dihydroquercetin (Figure 2b) indicates that the sensitivity coefficients of the same reagents and the order of their change are different for these two analytes. In the case of quercetin, the slope of calibration straight line increases in the series of reagents FeCl_3_ − 4-NPD − AgNO_3_ + NaOH, whereas in the case of dihydroquercetin, this series is the following: FeCl_3_ − AgNO_3_ + NaOH − 4-NPD. This is apparently due to the presence of a double bond, which stabilizes the π,π-conjugated system in the quercetin molecule. It promotes the formation of a complex with silver ion and causes its more efficient reduction, which, in turn, leads to a significant increase in signal associated with formation of silver nanoparticles.

The observations described above can be used to identify a flavonoid by its specific responses of the µPAD detection zones. This can be visualized as a 3D chart represented in Figure 3b. In this 3D chart, the coordinates of data points are the responses for three different reagents (AgNO_3_ + NaOH, 4-NPD, FeCl_3_), and the trends correspond to an increase in the flavonoid concentration. It can be seen from the figure that the straight lines diverge strongly in the 3D space. It allows for identifying flavonoids by considering which of the straight lines an experimental point measured for an analyzed sample belongs to. However, it should be emphasized that such identification is possible if only single analyte is present in the sample.

#### 3.2.2. Features of Merit

The analytical features of merit for the proposed µPAD are presented in Table 1. The limits of detection (LODs), which were calculated from 3s_0_ value, where s_0_ is standard deviation of a blank, lie in the range of 7.7–39 µg mL^−1^. FeCl_3_ (LOD = 39 μg mL^−1^) has the lowest sensitivity, and 4-NPD (LOD = 7.7 μg mL^−1^) has the highest one. The linearity ranges, with lower boundaries calculated from 10s_0_, are totally within an interval from 0.026 to 1.1 mg mL^−1^. Based on these data, it can be concluded that this µPAD is suitable for the determination of sufficiently high concentrations of flavonoids, which may be present, for example, in certain plant extracts or pharmaceutical preparations. In samples where the content of flavonoids is high, the µPAD may have some advantages over highly sensitive methods. The latter would require preliminary preparation and multiple dilution of the sample before the determination, which complicates the analysis procedure and may lead to significant systematic errors. At the same time, the absolute detection limits calculated for the µPAD (Table 1) are quite low, which is a consequence of its miniaturized design. It indicates the possibility of analyzing small amounts of samples, being another advantage of µPAD.

### 3.3. Determination of Dihydroquercetin Using the Three-Reagent µPAD and Digital Colorimetry

In addition to diffuse reflectance spectroscopy, the color change of samples can be recorded and quantified by digital colorimetry using a smartphone. However, the method of digital colorimetry often results in nonlinear calibration dependences described by an exponential equation [39,40]. An example of a calibration graph for the case of detecting dihydroquercetin by the reaction with 4-NPD (as a reagent providing the highest slope of a calibration curve) for all three color channels (R, G, and B) is shown in Figure S1 in the Supplementary Materials. The calibration curves can be successfully described by an exponential equation. These equations as well as features of merit for the determination of dihydroquercetin using digital colorimetry are given in Table 2. It can be seen that the sensitivity coefficients for various reagents for colorimetry change in a similar way to that for diffuse reflectance spectroscopy. For each detection zone, the most sensitive color coordinate can be found, which provides the lowest LOD. It is blue for the 4-NPD zone, red for the AgNO_3_ + NaOH zone, and red or green for the FeCl_3_ zone. However, generally, LODs achieved with a smartphone (ranging from 0.03 to 0.13 mg mL^−1^) appear higher than those calculated from the spectral measurements.

### 3.4. Selectivity and Analysis of Samples

Flavonoids are often determined in biologically active additives, pharmaceuticals, and vegetable raw materials; therefore, selectivity of the analysis relative to inorganic ions, mono- and polysaccharides, and ascorbic acid should be estimated. It was found that at least 1:100 excess of common inorganic ions (Na^+^, K^+^, Ca^2+^, SO_4_^2−^, NO_3_^−^, and Cl^−^) does not interfere with the determination of 0.25 mg mL^−1^ dihydroquercetin; the only exception is detection with AgNO_3_ + NaOH, which is affected by Cl^−^ already at 1:1 ratio. It is probably connected with the formation of AgCl on this detection zone, preventing the reduction of Ag^+^ to silver nanoparticles. This is an obvious limitation of using this sensing zone in the analysis of real samples. However, it should be noted that two other detection zones in the proposed three-reagent µPAD still can be applied in such cases.

Since the detection zone containing AgNO_3_ and NaOH is supposed to be the most subjected to interferences, additional effects of some organic interferents were assessed using this zone. It was shown that the determination is not affected by 1:100 excess of glucose and 1:1 amount of starch. However, it is affected by ascorbic acid already at 1:1 ratio because of the reduction of silver ions with this compound. As it was shown above, quercetin, which also belongs to the flavonoids class and possesses similar chemical properties, also provides responses of the µPAD test zones. This indicates certain limitation of selectivity inherent to methods of optical spectroscopy and colorimetry. However, a principle of multiple signal processing, illustrated above by the example of Figure 3b, together with big data mathematical analysis, can improve selectivity of the determination.

To check the accuracy of the determination using µPAD, the analysis of a food supplement “Digidrokvercetin” (Evalar) was performed. A tablet of the food supplement was carefully ground, and then dihydroquercetin was extracted with 50 mL of 100% ethanol in an ultrasonic bath for 30 min. Part of the extract was filtered, diluted 25 times with 100% ethanol, and used for the analysis. Reverse-phase HPLC was used as an alternative method for the determination of dihydroquercetin. The results are represented in Table 3. It can be seen that the determination with µPAD has acceptable accuracy and reproducibility.

### 3.5. Comparison with Other Methods

Comparison of the µPAD features of merit with published data is represented in Table 4. It indicates that the proposed method has reasonable LOD and broad linearity range. However, despite moderate sensitivity, the µPAD possesses improved selectivity due to the presence of three independent reagents in its composition. Another advantage of the proposed µPAD is ability to be easily redesigned in a required architecture, which stems from the concept of composable paper-based analytical devices discussed in this paper.

## 4. Conclusions

A three-reagent paper-based analytical device has been developed and proposed for the determination of dihydroquercetin. All the reagents, AgNO_3_ + NaOH, 4-NPD, and FeCl_3_, react with dihydroquercetin, utilizing different chemical principles, and produce colored products with different spectral characteristics. This is prospective for discriminating dihydroquercetin from other flavonoids. Both diffuse reflectance spectroscopy and digital colorimetry can be applied to detect analytical responses of the µPAD detection zones; however, the former method provides higher sensitivity. The proposed µPAD is applicable to analyze samples where the content of dihydroquercetin is quite high with their minimal dilution. The advantages of µPAD are the application of compact and inexpensive detection devices and low consumption of samples, reagents, and materials, which allows us to consider it a “green” method.

## Figures and Tables

**Table 1:** Analytical features of merit and calibration graphs equations for the determination of dihydroquercetin using the proposed µPAD and diffuse reflectance spectroscopy.

Detection Zone	Calibration Graph Equation	R^2^	Linearity Range, mg mL^−1^	LOD, mg mL^−1^	LOD, µg
4-NPD	ΔF = 3.53·C	0.978	0.026–1	0.0077	0.62
AgNO_3_ + NaOH	ΔF = 0.83·C	0.998	0.05–1	0.015	1.20
FeCl_3_	ΔF = 0.45·C	0.973	0.13–1.1	0.039	3.12

**Table 2:** Analytical features of merit and calibration graphs equations for the determination of dihydroquercetin using the proposed µPAD and digital colorimetry.

Detection Zone	Color Coordinate	Calibration Graph Equation	R^2^	Linearity Range, mg mL^−1^	LOD, mg mL^−1^	LOD, µg
4-NPD	Red	R = 190.3 + 35.4·exp(−c/0.27)	0.978	0.43–1	0.13	10
	Green	G = 163.8 + 47.5·exp(−c/0.32)	0.991	0.34–1	0.1	8.0
	Blue	B = 80.6 + 86·exp(−c/0.28)	0.999	0.11–1	0.03	2.4
AgNO_3_ + NaOH	Red	R = 153 + 45.4·exp(−c/0.19)	0.986	0.22–1	0.07	5.2
	Blue	B = 73.1 + 71.1·exp(−c/0.37)	0.971	0.34–1	0.1	8.0
FeCl_3_	Red	R = 160.3 + 70.3·exp(−c/0.44)	0.987	0.43–1	0.13	10
	Green	G = 140.1 + 78.2·exp(−c/0.60)	0.996	0.44–1	0.13	10

**Table 3:** Determination of dihydroquercetin using the proposed µPAD in a food supplement (detection with i1Pro2 mini-spectrophotometer, *n* = 3, *p* = 0.95).

Labeled Content, mg	µPAD			HPLC	
	Detection Zone	Found ± t_P,f_∙s/√n, mg	RSD, %	Found ± t_P,f_∙s/√n, mg	RSD, %
25	AgNO_3_	17 ± 11	26	29 ± 2	3
	4-NPD	37 ± 11	12		
	FeCl_3_	38 ± 8	9		

**Table 4:** Features of merit of µPAD-based methods for the determination of flavonoids and other polyphenols.

µPAD	Analytical Instrument	Analyte	Linearity Range, µg mL^−1^	LOD, µg mL^−1^	Reference
µPAD based on chemiluminescence of luminol/H_2_O_2_ system enhanced with cobalt-imidazole metal-organic framework	Smartphone	Gallic acid Quercetin Catechin Kaempferol Caffeic acid	0.5–50 1–100 1–100 2–100 2–120	0.12 0.28 0.46 0.85 1.23	[41]
µPAD based on producing silver nanoparticles	Mini-spectrophotometer	Quercetin Morin Dihydroquercetin	7–100 16–100 43–100	2.3 5.2 14	[32]
µPAD based on growth of gold and silver nanoparticles	Digital camera	Total polyphenol (hydroxytyrosol)	25–500	5 (Au) 6 (Ag)	[42]
µPAD based on luminescent graphene quantum dots embedded into nitrocellulose matrix	Smartphone	Quercetin	5–75	7.1; 20	[43]
Three reagent µPAD	Mini-spectro- photometer	Dihydroquercetin	26–1000	7.7; 15; 39	This study
µPAD based on iron tartrate	Table-top scanner	Total polyphenol (gallic acid)	0–1200	20	[44]
µPAD based on the Folin–Ciocalteu reagent	Smartphone	Total polyphenol	0–800	30 (µg g^−1^)	[45]

## Supplementary Materials

The following supporting information can be downloaded at: https://www.mdpi.com/article/10.3390/s22082893/s1, Figure S1: Colorimetric calibration graphs based on the interaction between 4-NPD and dihydroquercetin.
