# Supplementary material for: A Three-Reagent “Green” Paper-Based Analytical Device for Solid-Phase Spectrometric and Colorimetric Determination of Dihydroquercetin
Source: Sensors (Basel). 2022 Apr 9;22(8):2893. doi: 10.3390/s22082893 (PMC9030608; doi:10.3390/s22082893)
Supplement: Supplementary file 1 [file sensors-22-02893-s001.zip › sensors-1640979-supplementary.pdf]

Supplementary Material

Communication

A Three-Reagent "Green" Paper-Based Analytical Device for Solid-Phase Spectrometric and Colorimetric Determination of Dihydroquercetin

Vladimir V. Apyari [1,*], Aleksei A. Furletov [1], Vyacheslav I. Kalinin [1], Stanislava G. Dmitrienko [1] and Yury A. Zolotov [1,2]

[1] Department of Chemistry, Lomonosov Moscow State University, Leninskie Gory, 1/3, 119991 Moscow, Russia; aleksei_furletov@mail.ru (A.A.F.); workm2k@gmail.com (V.I.K.); dmitrienko@analyt.chem.msu.ru (S.G.D.); zolotov@analyt.chem.msu.ru (Y.A.Z.)
[2] Kurnakov Institute of General and Inorganic Chemistry, Russian Academy of Sciences, Leninsky Avenue, 31, 119991 Moscow, Russia
* Correspondence: apyari@mail.ru

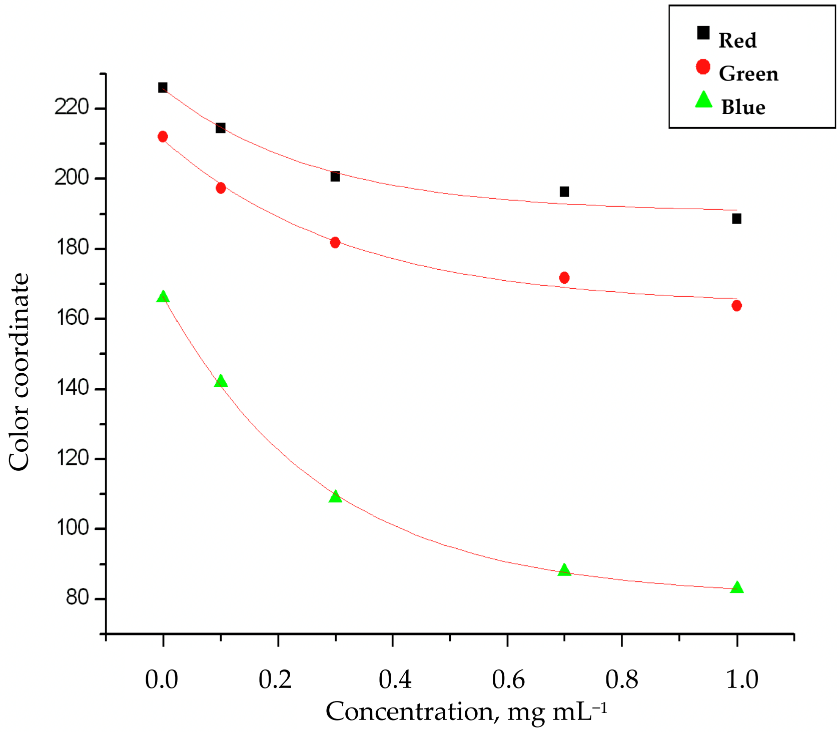

Figure S1. Colorimetric calibration graphs based on the interaction between 4-NPD and dihydroquercetin.
